# Global research trends of tumor microenvironment in non-small cell lung cancer with epidermal growth factor receptor mutation: a bibliometric analysis from 2014 to 2023

**DOI:** 10.3389/fimmu.2025.1555216

**Published:** 2025-03-20

**Authors:** Xiaoyan Chang, Chenghao Wang, Fei Wang, Linyou Zhang

**Affiliations:** ^1^ Department of Thoracic Surgery, Second Affiliated Hospital of Harbin Medical University, Harbin, China; ^2^ Department of Radiology, Second Affiliated Hospital, Harbin Medical University, Harbin, Heilongjiang, China

**Keywords:** non-small cell lung cancer, epidermal growth factor receptor, tumor microenvironment, data visualization, bibliometrics

## Abstract

**Purpose:**

Non-small cell lung cancer (NSCLC) is the most common type of lung cancer, and about half of the patients had mutations in the epidermal growth factor receptor (EGFR) gene. Changes in the tumor microenvironment after EGFR mutation are closely related to tumor progression and treatment efficacy.

**Materials and methods:**

We searched the Web of Science Core Collection database to select the articles related to tumor microenvironment in non-small cell lung cancer with epidermal growth factor receptor mutation. The countries/regions, institutes, authors, journals, references, and keywords were visualized and analyzed.

**Results:**

227 relevant studies were obtained from WoSCC. These articles came from 102 countries and 1179 institutions. After network analysis, it was found that the intensity of USA cooperation with China was the greatest (LS=13), followed by cooperation with South Korea (LS=3) and with Japan (LS=3). A total of 2267 authors participated the all 227 articles. 112 journals were covered, and *Frontiers in Oncology* published most papers (n=16, 14.3%). A total of 7964 co-cited references are related to TME in NSCLC with EGFR mutation. “EGFR” is the keyword with the highest centrality (C=0.31) and first appeared. The keywords that burst in the last 1 year (2022-2023) are “immunotherapy”, “mechanism”, “lung neoplasms”, “T cells”, and “multicenter”.

**Conclusion:**

Effective drug treatment of advanced NSCLC with EGFR mutations after failure of first-line chemotherapy is one of the hotspots, in which the efficacy of immune checkpoint inhibitors may be the direction of the current and future studies that need to find a breakthrough.

## Introduction

Lung cancer is the most common malignant tumor in terms of both incidence and mortality (1). Non-small cell lung cancer (NSCLC) is the most common type of lung cancer, accounting for 80%-85% (2). Activating mutations in the epidermal growth factor receptor (EGFR) gene occur in at least 50% of Asian NSCLC patients (3). The high aggressiveness of NSCLC and the lack of effective early screening programs have led to 70% of lung cancer patients in China being diagnosed at advanced stages (4). Targeted therapy and immunotherapy are effective therapeutic options for the treatment of advanced NSCLC. Tyrosine kinase inhibitors (TKIs) significantly prolong progression-free survival in EGFR mutation-positive NSCLC patients (5). Multiple clinical guidelines recommend TKIs-based regimens as the preferred first-line treatment for patients with EGFR mutation-positive advanced NSCLC (6, 7). However, patients inevitably develop drug resistance during TKIs targeted therapy and there is no consensus on subsequent treatment. Immune checkpoint inhibitors (ICIs) are another effective treatment option for patients with advanced NSCLC, able to increase patients’ 5-year survival by up to 30% (8).

Tumor cells, stroma, immune cells, and extracellular components together constitute the tumor immune microenvironment (TME), a complex and dynamic ecosystem, which is the “soil” for tumor survival (9). TME plays an important role in tumor immunosuppression, drug resistance, tumor invasion, and metastatic growth (10). EGFR mutations suppress the tumor immune microenvironment by interfering with multiple intracellular pathways and regulating immune helper cells (11). It has been suggested that the survival benefit of ICIs is only for the first-line treatment of advanced driver-negative NSCLC, while the efficacy remains limited when comparing EGFR mutation-positive patients (5, 12). The complex immune microenvironment may be one of the main reasons for poor efficacy.

To our knowledge, there are few studies on the particular scientometrics of knowledge mapping of publications of TME in NSCLC with EGFR mutation. Bibliometric analysis is a literature analysis method that describes and discovers current trends and topics in a certain area, and emphasizes relationships using mathematical and statistical techniques (13), which is an advantage that traditional experimental or clinical research approaches cannot provide. This study evaluates the articles about tumor microenvironment in NSCLC with EGFR mutation from January 2014 to December 2023 to characterize the present status of research and hotspots, as well as to provide a reference for this field that needs to be optimized and improved in the future.

## Materials and methods

### Source of data and search strategy

All included articles in this study were retrieved from the Web of Science Core Collection (WoSCC). The search terms are detailed in the online resource

**Supplementary Table S1**. The deadline for search runs until August 02, 2024. The inclusion criteria were as follows: 1) the publication types were only “article”; 2) the language was limited to English. The exclusion criteria included: 1) other publication types, such as reviews, editorial materials, meeting abstracts, early access, etc.; 2) repeat or retract study.

The institutional review board of the hospital deemed that ethical approval was not necessary, as this was a pure bibliometric study.

### Data collection and analysis

Two authors independently searched the WoSCC database for relevant literature, and exported it in TXT format of “full records and references”. The citation report was retrieved from WoSCC including citing articles, times cited, average per item, and H-index. In addition, impact factors (IF) and quartiles of journal categories were obtained from Journal Citation Reports (JCR) 2021. If there are disagreements, the opinions are harmonized by discussion or a third author.

### Statistical methods

Microsoft Office Excel 2019 (Microsoft, Redmond, Washington, USA) was used to plot the trend of total publications, and a polynomial regression model was built to predict the number of studies published in the next 1-2 years, and the goodness of fit (R^2^) was used to assess the degree of fit of the regression line to the observed values. The closer the value of R^2^ is to 1, the better the fit is. VOSviewer (version 1.6.19) was used to explore collaborative networks between countries or regions/institutions/authors/journals. Scimago Graphics (1.0.32) was used to map the cooperation between various countries/regions. CiteSpace (Chaomei Chen, Drexel University, USA, version 6.1.R 6) was used to extract keywords and references from publications with high citation bursts and build a double map overlay for journals, to identify current research hotspots and frontiers (14, 15). In our study, CiteSpace was used to analyze the keywords’ co-occurrence, keywords clustering, keywords timeline, keyword bursts, and references. Details of the parameter settings are in the **Supplementary Material**.

## Results

### Annual growth trend of publications

**Figure 1:**
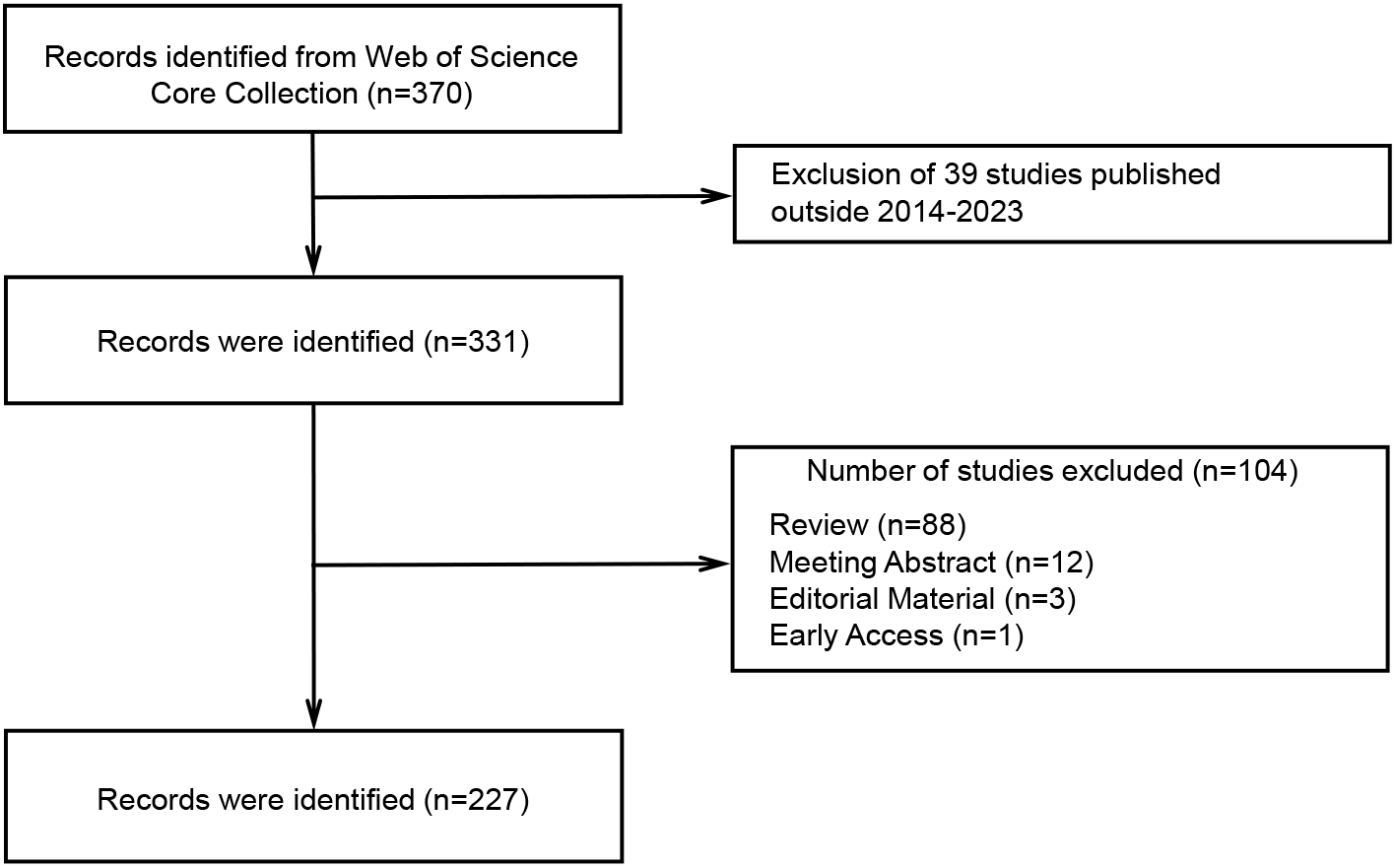
The Flowchart depicting the study’s selection process.

**Figure 2:**
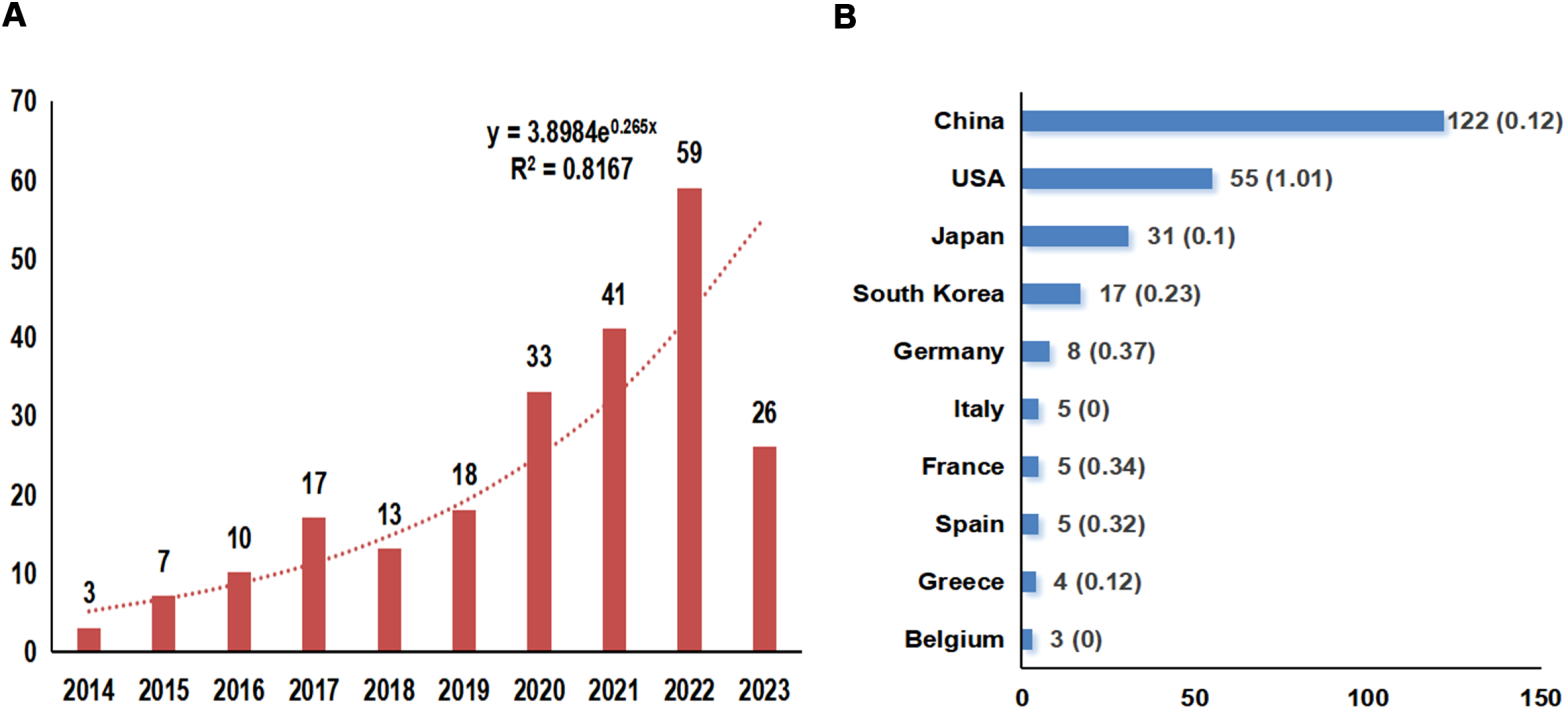
Overview of annual publication numbers and the polynomial curve fitting of publication growth **(A)** and the Top 10 countries of the publication numbers **(B)**. The horizontal coordinate indicates the year of the studies published and the vertical coordinate indicates the number of publications, and an exponential formula was constructed to predict the number of publications in the field in the next 1-2 years; R^2^ is the coefficient which is a measure of how closely the regression line fits the observations, the higher the value of R^2^, the better the curve is fitted.

According to the search strategy, 370 relevant studies were obtained from WoSCC. After screening by temporal conditions, a total of 331 studies were published between 2014 and 2023, including 227 articles, 88 reviews, 12 meeting abstracts, 3 editorial materials, and 1 early access (**Figure 1**). Therefore, a final total of 227 articles were included in the subsequent analysis. The total times cited in these studies were 8424, the average per item of citations was 37.11, and the H-index was 45. We constructed a polynomial formula based on the number of studies from 2014-2023: y = 3.8984e^0.265x^, and R² was 0.8167 (**Figure 2A**). Using this formula for predicting the number of studies in 2023 and 2024, approximately 72 and 94 articles associated with TME in NSCLC with EGFR are expected to be published. But in fact, only 26 articles were published in 2023.

### Countries/regions and institutions analysis

**Figure 3:**
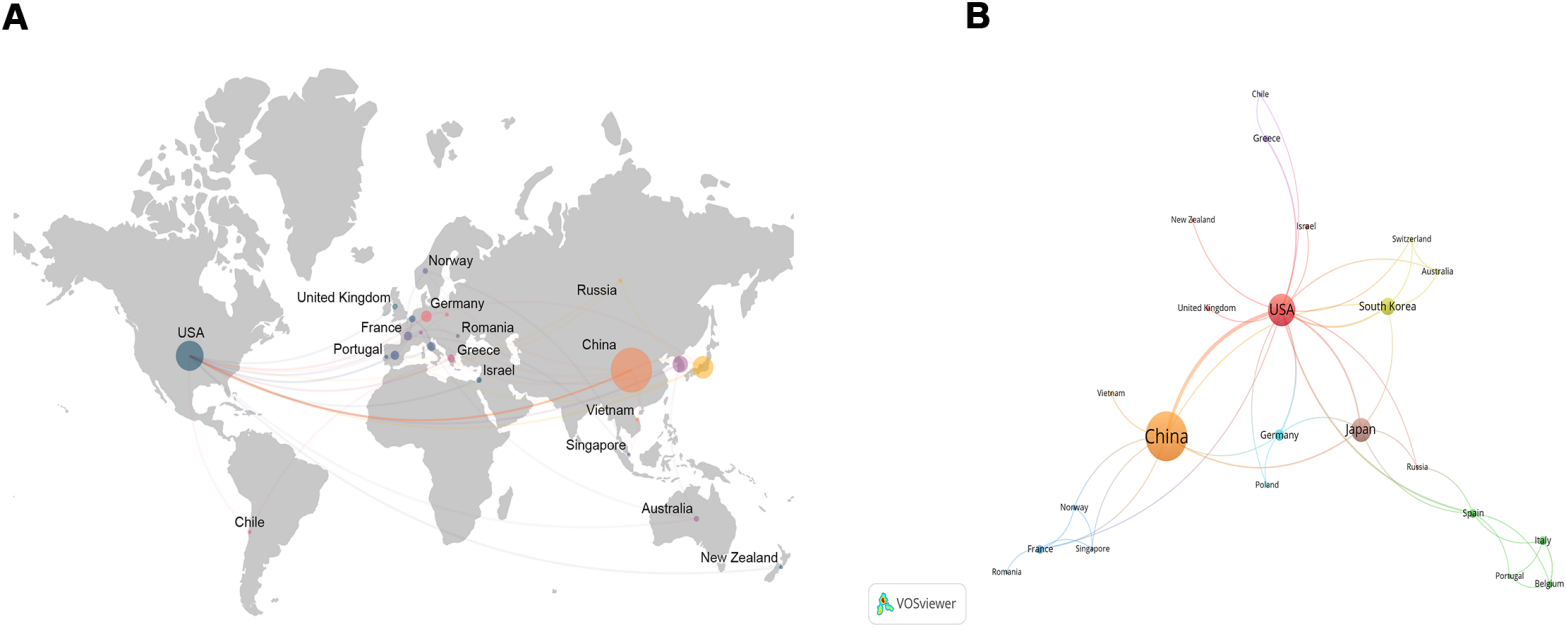
The network map about the status of global research **(A)** and the intensity of collaboration **(B)**. The larger the circle, the greater the number of publications, and the thicker the curve, the closer the cooperation.

These articles came from 102 countries and 1179 institutions. The top 3 countries in terms of the number of articles are China (n=122, 53.7%), the United States of America (USA) (n=55, 24.2%), and Japan (n=31, 13.7%), respectively (**Supplementary Table S2**). Among the top 10 countries, the centrality (C) of the USA is the highest (C=1.01), followed by Germany (C=0.37) and France (C=0.34), respectively (**Figure 2B**). After network analysis, it was found that the intensity of USA cooperation with China was the greatest [Link strength (LS)=13], followed by cooperation with South Korea (LS=3) and with Japan (LS=3) (**Figures 3A, B**). For institutions, the institutions that published the most number of articles are Harvard University (n=11), Shanghai Jiao Tong University (n=11), and Fudan University (n=11), respectively (**Table 1**). Among the top 10 institutions, the centrality of the University of Texas System is the highest (C=0.43), followed by Harvard University (C=0.38) and Tongji University (C=0.35), respectively.

**Table 1:** The top 10 institutions published articles related to tumor microenvironment in non-small cell lung cancer with epidermal growth factor receptor mutation.

Institution	Location	Counts	Centrality
Harvard University	USA	11	0.38
Shanghai Jiao Tong University	China	11	0.33
Fudan University	China	11	0.02
Chinese Academy of Sciences	China	9	0.07
Harvard Medical School	USA	6	0.23
National Cancer Center - Japan	Japan	6	0.16
Tongji University	China	6	0.35
Chinese Academy of Medical Sciences - Peking Union Medical College	China	6	0.02
Central South University	China	5	0.08
University of Texas System	USA	5	0.43

### Authors and co-cited authors

**Figure 4:**
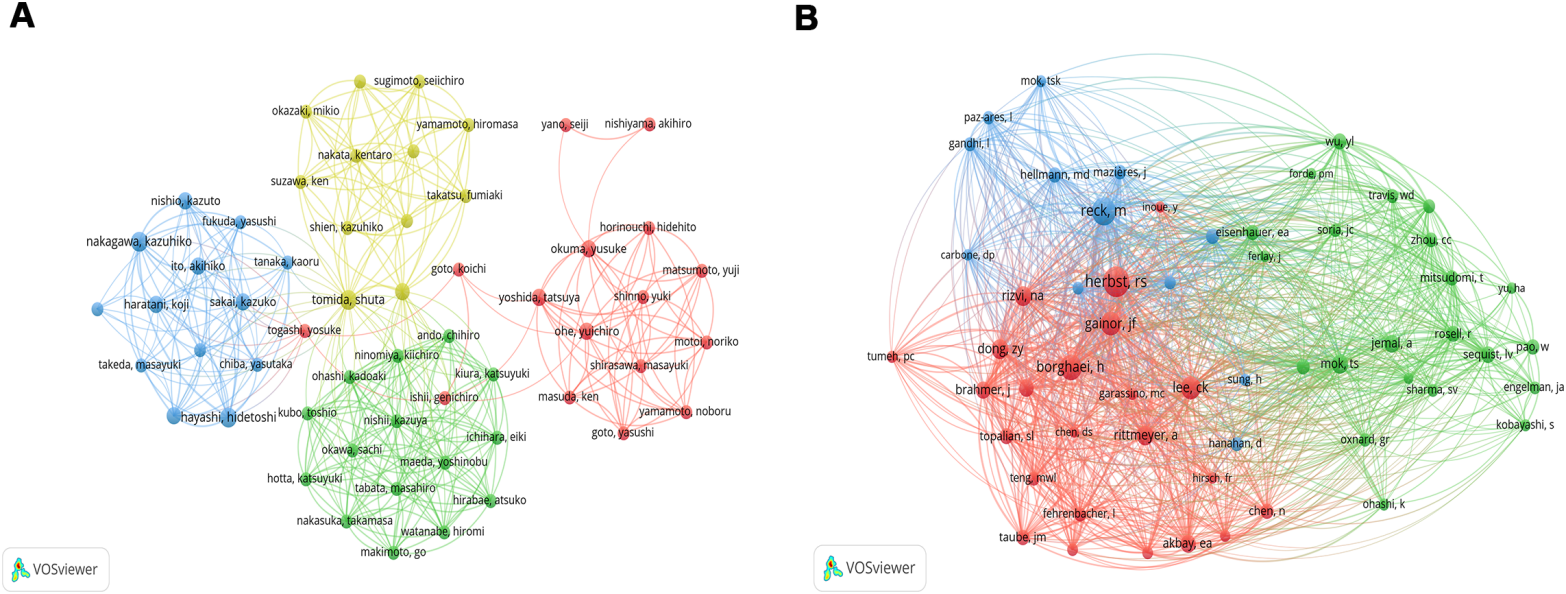
The network map of co-authorship **(A)** and co-citation authors **(B)**. The circle indicates the author, the color indicates the same cluster, and the larger the circle, the larger the weight; the thicker the lines, the larger the link’s strength (The subsequent figures’ notes are similar).

A total of 2267 authors participated the all 227 articles. Among the top 10 authors in terms of the number of publications, Tomida Shuta, Hayashi Hidetoshi, and Nakagawa Kazuhiko published 4 articles, and Toyooka Shinichi, Haratani Koji, Tanizaki Junko, Ito Akihiko, Ohe Yuichiro, Yoshida Tatsuya, and Nishio Kazuto published 3 articles (**Table 2**). Among them, Hayashi Hidetoshi and Nakagawa Kazuhiko have the highest number of citations (n=247). Tomida Shuta has the highest total link strength [Total link strength (TLS) =48], followed by Toyooka Shinichi (TLS=38), Hayashi Hidetoshi (TLS=32), and Nakagawa Kazuhiko (TLS=32, **Figure 4A**). For co-cited authors, the top 3 cited authors are Herbst RS (Citation=76), Reck M (Citation=64), and Borghaei H (Citation=53) (**Figure 4B**).

**Table 2:** The top 10 authors and co-cited authors about tumor microenvironment in non-small cell lung cancer with epidermal growth factor receptor mutation.

Authors	Counts	Citation	Average Citation	Total link strength	Co-cited authors	Co-Citation
Tomida, Shuta	4	164	41	48	Herbst, RS	76
Hayashi, Hidetoshi	4	247	61.75	32	Reck, M	64
Nakagawa, Kazuhiko	4	247	61.75	32	Borghaei, H	53
Toyooka, Shinichi	3	21	7	38	Gainor, JF	47
Haratani, Koji	3	213	71	26	Lee, CK	44
Tanizaki, Junko	3	213	71	26	Dong, ZY	34
Ito, Akihiko	3	209	69.6667	24	Rittmeyer, A	34
Ohe, Yuichiro	3	109	36.3333	23	Rizvi, NA	33
Yoshida, Tatsuya	3	109	36.3333	23	Mok, TS	31
Nishio, Kazuto	3	104	34.6667	22	Akbay, EA	27

### Journals and co-cited academic journals

**Figure 5:**
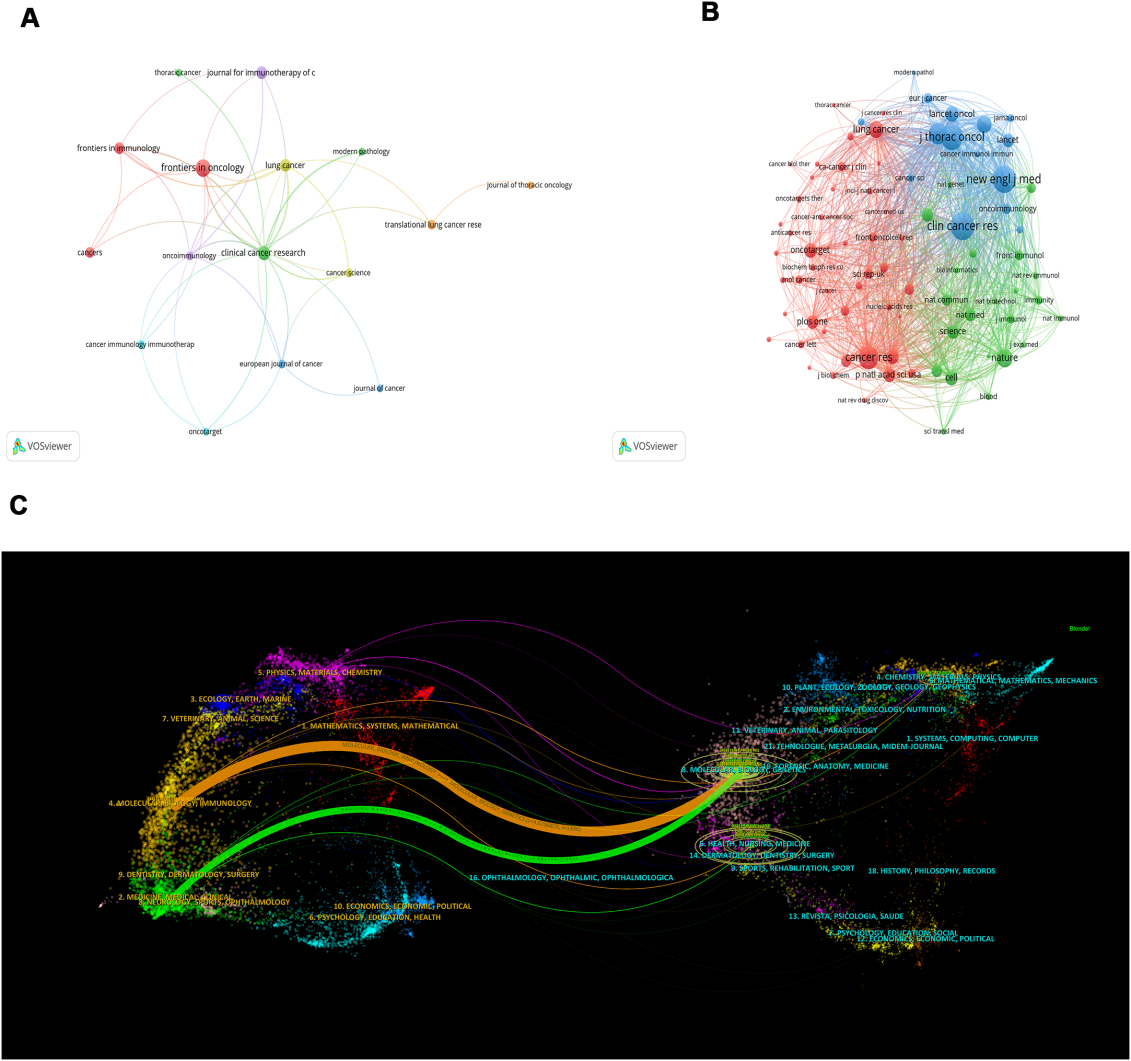
A network map of the journal **(A)**, co-citation journal **(B)**, and a dual-map overlap **(C)** of academic journals with studies researching tumor microenvironment in non-small cell lung cancer with epidermal growth factor receptor mutation. In **Figure 5C**, the citing journal is on the left, and the cited journal is on the right, with the citation relationship indicated by the colored path. The links to the left and right journals reflect citation relationships.

Of the 227 articles related to the tumor microenvironment after EGFR mutations in non-small cell lung cancer, 112 journals were covered (**Table 3**), *Frontiers in Oncology* published most papers (n=16, 14.3%), followed by *Clinical Cancer Research* (n=11, 9.8%), *Lung Cancer* (n=9, 8.0%), and *Journal for Immunotherapy of Cancer* (n=9, 8.0%). Among the top 10 journals, the journal with the highest impact factor is the *Journal for Immunotherapy of Cancer* (IF=10.3), followed by *Clinical Cancer Research* (IF=10) and the *European Journal of Cancer* (IF=7.6). We mapped the network of journals and found that *Clinical Cancer Research* collaborated most actively with other journals (TLS=40), followed by *Lung Cancer* (TLS=19) and *Oncoimmunology* (TLS=19) (**Figure 5A**).

**Table 3:** The Top 10 journals and co-cited journals related to tumor microenvironment in non-small cell lung cancer with epidermal growth factor receptor mutation.

Journals	Counts (n=112)	IF (2023)	JCR	Total link strength	Co-cited journals	Co-citations	IF (2023)	JCR	Total link strength
Frontiers in Oncology	16 (14.3%)	3.5	Q2	12	Clinical Cancer Research	451	10	Q1	14667
Clinical Cancer Research	11 (9.8%)	10	Q1	40	New England Journal of Medicine	431	96.2	Q1	13769
Lung Cancer	9 (8.0%)	4.5	Q1	19	Journal of Thoracic Oncology	396	21	Q1	13818
Journal for Immunotherapy of Cancer	9 (8.0%)	10.3	Q1	7	Cancer Research	331	12.5	Q1	10397
Frontiers in Immunology	8 (7.1%)	5.7	Q1	12	Journal of Clinical Oncology	290	42.1	Q1	10004
Cancers	7 (6.3%)	4.5	Q1	3	Nature	223	50.5	Q1	7975
Translational Lung Cancer Research	6 (5.4%)	4	Q1	3	Annals of Oncology	206	56.7	Q1	7069
Oncoimmunology	5 (4.5%)	6.5	Q1	19	Lung Cancer	201	4.5	Q1	7088
European Journal of Cancer	5 (4.5%)	7.6	Q1	8	Lancet Oncology	174	41.6	Q1	6126
Cancer Science	5 (4.5%)	4.5	Q1	9	Cell	153	45.5	Q1	5571

In the analysis of co-cited journals, we found that the top 3 co-cited journals are *Clinical Cancer Research* (Co-citation=451), *New England Journal of Medicine* (Co-citation=431), and *Journal of Thoracic Oncology* (Co-citation=396). Among the top 10 co-cited journals, the impact factor of the *New England Journal of Medicine* (IF=96.2) is the highest, followed by the *Annals of Oncology* (IF=56.7) and *Nature* (IF=50.5). Similarly, the network mapping of co-cited journals revealed that *Clinical Cancer Research* (TLS=14667), *New England Journal of Medicine* (TLS=13769), *Journal of Thoracic Oncology* (TLS=13818), *Cancer Research* (TLS=10397), and *Journal of Clinical Oncology* (TLS=10004) are the most active in cooperating with other journals (**Figure 5B**). After plotting the dual-map overlay of the journals, we obtained two paths (**Figure 5C**). The two paths represent the articles published in molecular/biology/genetics journals that are mainly cited by literature in molecular/biology/immunology and medicine/medical/clinical journals.

### Co-cited references

**Figure 6:**
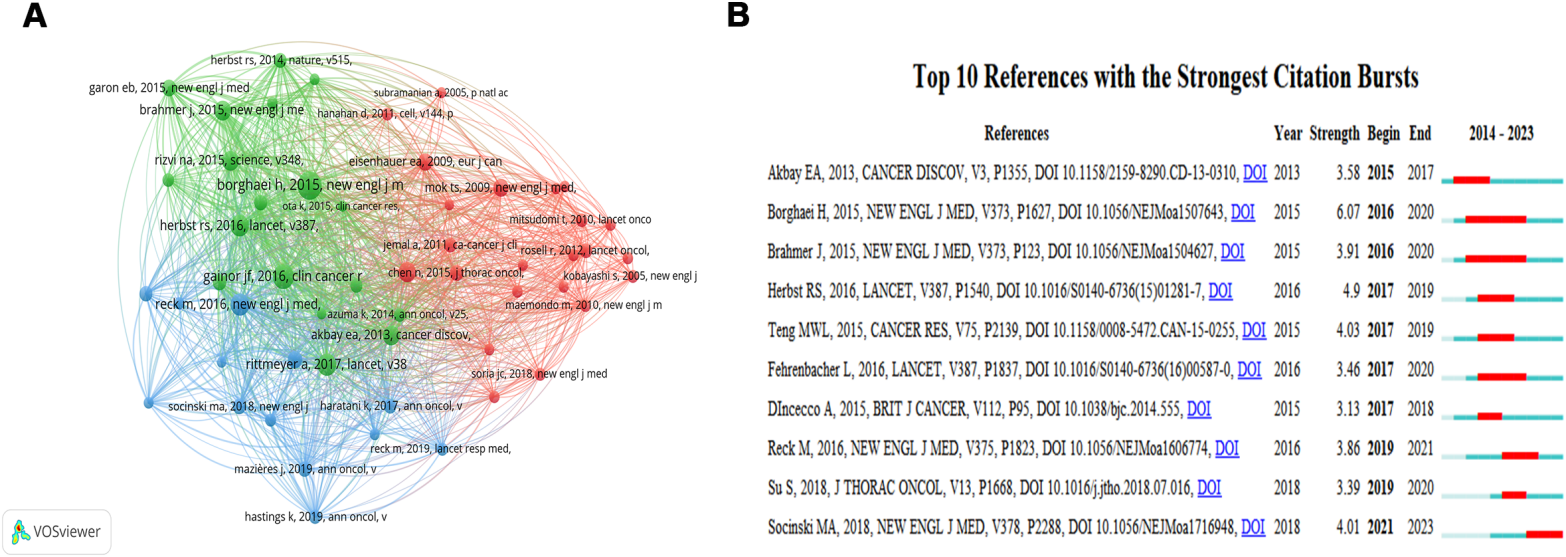
The network map of cited references **(A)** and visualization map of the Top 10 references with the strongest citation bursts **(B)**. In **Figure 6B**, “Begin” indicates the time of keyword bursts, “End” indicates the time of keyword bursts, and “Strength” indicates the strength of keyword bursts. The strength of the keyword bursts is proportional to the influence of the keyword.

A total of 7964 co-cited references are related to TME in NSCLC with EGFR mutation. The co-citation reference with the highest citations is “Borghaei H, 2015, NEW ENGL J MED, V373, P1627” (Citation=52), followed by “Gainor JF, 2016, CLIN CANCER RES, V22, P4585” (Citation=40), “Rittmeyer A, 2017, LANCET, V389, P255” (Citation=33), and “Reck M, 2016, NEW ENGL J MED, V375, P1823” (Citation=33) (**Figure 6A**, **Supplementary Table S3**). A burst analysis of these co-cited references revealed that the burst strength of the top 10 references ranged from 3.13 to 6.07 (**Figure 6B**). The reference with the strongest citation burst was “Borghaei H, 2015, NEW ENGL J MED, V373, P1627” (Strength=6.07), which title was “Nivolumab versus Docetaxel in Advanced Nonsquamous Non–Small-Cell Lung Cancer” with citation bursts from 2016 to 2020.

### Analysis of keywords co-occurrence, clusters, timeline, and bursts

**Figure 7:**
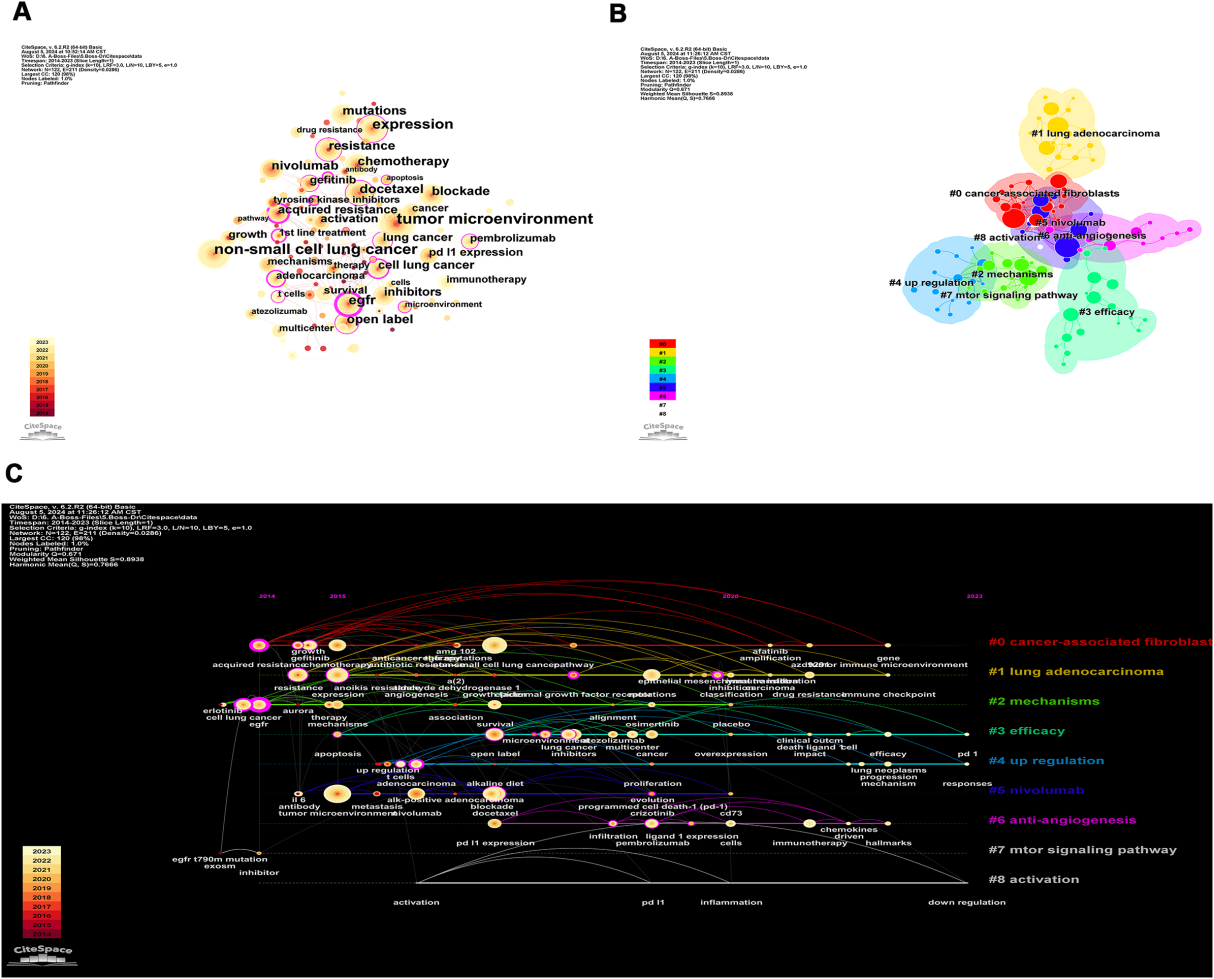
The network of keywords co-occurrence in 227 studies related to tumor microenvironment in non-small cell lung cancer with epidermal growth factor receptor mutation. **(A)** Keyword co-occurrence. The size of the nodes reflects the number of publications; the purple circle represents centrality, and the thicker the circle means higher centrality; the more and thicker connecting lines between the nodes mean closer cooperation; **(B)** Keyword cluster. Different color blocks in the clustering knowledge map represent different clusters, and the nodes contained in the color blocks all belong to the same cluster; **(C)** Keyword timeline. Each timeline corresponds to each cluster, and the keywords on the timeline appear in chronological order from left to right.

Using CiteSpace for keywords co-occurrence, a total of 122 nodes and 211 lines were obtained (**Figure 7A**). **Table 4** shows the top 10 high-frequency keywords related to TME in NSCLC with EGFR mutation were in order: “tumor microenvironment” (n=76), “non-small cell lung cancer” (n=66), “expression” (n=52), “EGFR” (n=40), “docetaxel” (n=36), “chemotherapy” (n=33), “blockade” (n=33), “resistance” (n=33), “nivolumab” (n=31), and “mutations” (n=31). Among these keywords, “EGFR” has the highest centrality (C=0.31), followed by “expression” (C=0.22) and “resistance” (C=0.20). In the keyword cluster analysis mapping (**Figure 7B**), the cluster module value Q = 0.671 indicates a significant cluster structure, and the average cluster profile value S = 0.8938 indicates a convincing clustering result. A total of 9 clusters were obtained, including “#0 cancer-associated fibroblasts (cafs)”, “#1 lung adenocarcinoma”, “#2 mechanisms”, “#3 expression”, “#4 up-regulation”, “#5 nivolumab”, “#6 anti-angiogenesis”, “#7 mtor signaling pathway”, and “#8 activation”.

**Table 4:** The top 10 most frequent keywords related to tumor microenvironment in non-small cell lung cancer with epidermal growth factor receptor mutation.

Keywords	Counts	Centrality
tumor microenvironment	76	0.12
non-small cell lung cancer	66	0.06
expression	52	0.22
egfr	40	0.31
docetaxel	36	0.06
chemotherapy	33	0.03
blockade	33	0.08
resistance	33	0.20
nivolumab	31	0.05
mutations	31	0.07

**Figure 8:**
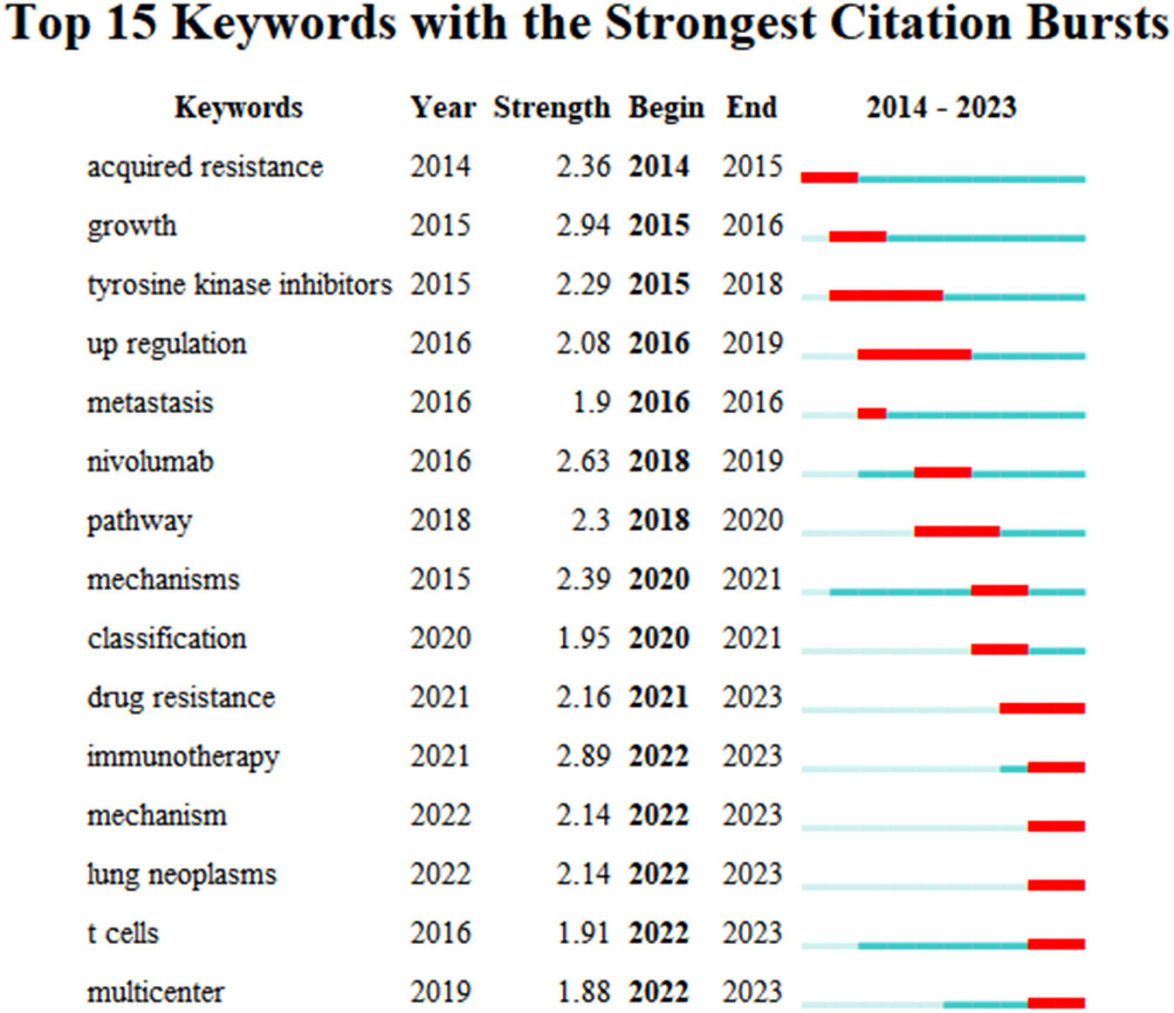
The top 15 keywords with strong citation bursts related to artificial intelligence applied in the field of hepatocellular carcinoma during 2014.01-2023.12.

Based on these clusters, we conducted a timeline analysis (**Figure 7C**). The figure showed that each cluster corresponds to a timeline where keywords appear sequentially over time from left to right (2014-2023). In **Figure 7C**, the keywords that first appeared in 2014 are “acquired resistance”, “EGFR”, and “inhibitor”, and the latest keywords appearing in 2023 are “PD-L1”, “responses”, and “down-regulation”. Based on the keyword burst analysis we found that in the top 15 keywords, the keyword with the highest strength is “growth” (Strength=2.94), followed by “immunotherapy” (Strength=2.89) and “nivolumab” (Strength=2.63). The keywords that burst in the last 1 year (2022-2023) are “immunotherapy”, “mechanism”, “lung neoplasms”, “T cells”, and “multicenter” (**Figure 8**).

## Discussion

A total of 227 articles related to non-small cell lung cancer with concomitant EGFR mutations and its tumor microenvironment were searched in the last decade. The exponential increase in the number of publications per year from 2014 to 2022 indicates that this field is one of the hotspots of scientific research. However, there is a decrease in the number of publications in 2023 compared to the expected amount, possibly due to the outbreak and continuation of the New Crown epidemic, which has hampered the smooth running of basic experiments globally. Globally, China is the most active in this field of research. First of all, China is one of the most populous countries in the world, and the annual incidence of respiratory cancers in China is about 1,060,584, accounting for 42.8% of the global incidence, and the annual deaths of respiratory cancers are about 733,291, accounting for 40.4% of the global deaths. China leads in both morbidity and mortality (1). Although it is China that occupies the first place in terms of the number of publications, the USA has the highest degree of centrality, and its high centrality demonstrates its preeminence and influence in the field. In addition, the United States has been most active in cooperating with Asian countries, with China being the most closely cooperating country, followed by South Korea and Japan. Similarly, the top three institutions in terms of issuing institutions are Harvard University from the United States, Shanghai Jiao Tong University, and Fudan University from China. Among them, Harvard University has the highest degree of centrality. Harvard’s medical disciplines have a world-class academic impact, with Harvard Medical School being the top medical school in the world. The co-citation reference with the highest citations and strength is “Borghaei H, 2015, NEW ENGL J MED, V373, P1627” (Citation=52) and (Strength=6.07), which title was “Nivolumab versus Docetaxel in Advanced Nonsquamous Non–Small-Cell Lung Cancer” with citation bursts from 2016 to 2020 (16). Effective drug therapy after failure of first-line chemotherapy in advanced NSCLC is one of the hot topics that have been focused on in the field.

In NSCLC, EGFR is the most common mutation besides KRAS (17), especially in patients with advanced NSCLC. It is reported that (18), Asian populations exhibit higher rates of EGFR mutations and lower rates of KRAS Gly12Cys mutations. EGFR mutations in NSCLC include “classic” EGFR mutations (~85%) and rare EGFR mutations (~15%) (3, 19). Previous studies show EGFR and ALK mutations significantly inhibit ICIs efficacy (20, 21). The impact o mutations on patient prognosis and treatment efficacy is not dominated by a single mutation, but involves complex interactions between multiple mutations (22–24). Patients with EGFR-mutated NSCLC are also commonly comorbid with other mutations, and for patients with EGFR-mutated NSCLC, the modulatory effect of EGFR mutations on the efficacy of immunotherapy is also likely to be affected by the inclusion of other mutations (25, 26). However, the impact of EGFR co-mutations on the efficacy of ICIs is currently unknown. Through this bibliometric analysis, we can see that immunotherapy research for NSCLC with EGFR mutations has been a hot spot of research in recent years, in which the therapeutic target is mainly focused on PD-L1. PD-L1 is a cell-surface protein found in a wide range of cell types, including immune cells and some types of tumor cells, which inhibits the immune response by binding to PD-1 and inhibiting the activation and proliferation of T cells (27). It has been shown that the level of PD-L1 expression in NSCLC can be a predictor of the efficacy of ICIs (28). However, in NSCLC patients with concomitant EGFR mutations, ICIs have very limited efficacy as monotherapy. Some studies even suggest that the immunosuppressive effect mediated by EGFR mutations may be an important reason for limiting the efficacy of ICIs, i.e., EGFR mutant NSCLC recruits immunosuppressive cells by secreting cytokines to repel anti-tumor immune cells, thus creating an immune-silencing microenvironment (11, 29, 30). However, “nivolumab” is a fully human IgG4 PD-1 immune checkpoint inhibitor antibody, and disrupts PD-1-mediated signaling and may restore antitumor immunity (16). Similarly, this is consistent with our findings. Nivolumab became the first FDA-approved drug for ICIs for patients with squamous NSCLC whose disease progressed during or after platinum-based chemotherapy on March 4, 2015, and has subsequently been designated for patients with non-squamous NSCLC as well (31). The CheckMate 026 trial compared the efficacy of nivolumab monotherapy to chemotherapy for the first-line treatment of patients with PD-L1-positive (≥1%) stage IV or recurrent NSCLC and showed that nivolumab did not result in a significant prolongation of survival versus chemotherapy in patients with PD-L1 expression levels of 5% or higher (32). In addition to nivolumab, pembrolizumab, and atezolizumab have also shown high anti-tumor efficacy. However, further studies are needed to determine which ICIs to choose for monotherapy and which ICIs to combine with chemotherapy regimens have the best efficacy and the weakest adverse effects. In addition, biomarkers that can be used as predictors of NSCLC immunotherapy efficacy include: tumor mutation burden as a predictor of efficacy for nivolumab mono- or combination immunotherapy for NSCLC (33); the DNA mismatch repair system, which recognizes and repairs nucleotide base mismatches and is a key guardian of genome integrity and can be used as a discriminator of microsatellite stability to predict prognosis (34), et al. Additionally, circulating tumor DNA (ct DNA) is a DNA fragment from the tumor genome in the peripheral blood that carries the characteristics of a defined tumor and is released into the peripheral blood through tumor necrosis, apoptosis, or active secretion, and the plasma concentration of which is closely related to tumor size and stage (35). The ctDNA half-life in non-small cell lung cancer is short, so theoretically multiple plasma ctDNA dynamic assays could reflect the dynamic changes in tumor load *in vivo* over time (36).

### Limitations

In this study, several limitations are present in the bibliometric analysis. Firstly, the data of this study are only from the WoSCC database, and other databases are ignored, which may miss some relevant studies. In the future, we will incorporate more databases, such as Scopus, PubMed, and Google Scholar, to obtain more complete literature and citation information and to enhance the reliability of the study results. Secondly, we only analyzed NSCLC patients with concomitant EGFR mutations and did not analyze in depth whether there was a difference in study trends for specific pathologic subtypes.

## Conclusion

In summary, the results of previous studies as well as the present study indicate that in future studies, exploring factors influencing immunotherapy resistance in NSCLC with EGFR mutations (e.g., EGFR mutation subtype, combined with other mutations, etc.), novel immunotherapeutic methods (e.g., chimeric antigen receptor T cells, tumor-infiltrating lymphocyte therapy), changes in the TME before and after immunotherapy, and the prediction of immunotherapy efficacy are still hot topics and bottlenecks. Although promising results have been obtained in some clinical trials, there is still a need for more and longer trials for validation to improve the stability of the results, especially international, multicenter, and prospective validation.

## Data Availability

The original contributions presented in the study are included in the article/**Supplementary Material**. Further inquiries can be directed to the corresponding author.
