# Supplementary material for: Global research trends of tumor microenvironment in non-small cell lung cancer with epidermal growth factor receptor mutation: a bibliometric analysis from 2014 to 2023
Source: Front Immunol. 2025 Mar 20;16:1555216. doi: 10.3389/fimmu.2025.1555216 (PMC11965360; doi:10.3389/fimmu.2025.1555216)
Supplement: Supplementary file 1 [file Table1.docx]

**Global Research Trends of Tumor Microenvironment in Non-Small Cell Lung Cancer with Epidermal Growth Factor Receptor Mutation: A Bibliometric Analysis from 2014 to 2023**

[Statistical Methods 1](#_Toc21197)

[Table S 2](#_Toc20116)

# Statistical Methods

The parameters of CiteSpace were set as follows: 1) time slicing was from 2014.01 to 2023.12, and the time cut-off point of analysis was 1 year; 2) all options in the term source were selected; 3) for node types, selected country, institution, author, and keyword respectively to conduct visual analysis and generate co-occurrence map, and the threshold was used to screen the Top N authors with the highest frequency in each time slice (n=10), highlighting important literature; 4) selection criteria: g-index, g2 ≤ k∑i≤gci, k ∈ Z+,k = 10); 5) pruning: selected pathfinder, pruning sliced networks, and pruning the merged network in the pruning function area to highlight the connection diagram of important nodes. We used centrality to evaluate the influence and importance of keywords, and both the modularity (Q value) and silhouette (S value) to evaluate the degree of keyword clustering. It is generally believed that clustering is reasonable if Q > 0.3 and S > 0.5, and S > 0.7 means that clustering is convincing. In VOSviewer, nodes are used to represent the items, and their size is determined by their co-occurrence frequency in titles and abstracts. We selected the minimum number of documents of the nodes according to the needs of data visualization and set other documents as the default value. Total link strength (TLS) indicates the total strength of the cooperation links of a given item with other items. Links strength (LS) indicates the strength of cooperation links of a given item with another item.

# Table S

# Table S1. Search Query with Topic Search

| N | Topic Search | Results (N) |
| --- | --- | --- |
| #1 | “Non-small cell lung cancer” OR “Non-Small-Cell Lung Carcinomas” OR “Non-Small-Cell Lung Carcinoma” OR “Non Small Cell Lung Carcinoma” OR “Nonsmall Cell Lung Cancer” OR “Non-Small Cell Lung Carcinoma” | 81289 |
| #2 | **EGFR OR “epidermal growth factor receptor”** | 93147 |
| #3 | **Microenvironment** | 134994 |
| Total | #1 AND #2 AND #3 | 370 |

Table S2. The top 10 productive countries/regions related to tumor microenvironment in non-small cell lung cancer with epidermal growth factor receptor mutation.

| Rank | Country/region | Article counts | Citations | Links | Total link strength | Centrality |
| --- | --- | --- | --- | --- | --- | --- |
| 1 | China | 122 | 2983 | 8 | 21 | 0.12 |
| 2 | USA | 55 | 3613 | 15 | 37 | 1.01 |
| 4 | Japan | 31 | 1293 | 6 | 9 | 0.1 |
| 3 | South Korea | 17 | 368 | 5 | 7 | 0.23 |
| 5 | Germany | 8 | 458 | 4 | 6 | 0.37 |
| 6 | Spain | 5 | 1033 | 6 | 8 | 0 |
| 7 | Italy | 5 | 331 | 3 | 4 | 0.32 |
| 8 | France | 5 | 297 | 5 | 5 | 0.34 |
| 9 | Greece | 4 | 85 | 2 | 4 | 0.12 |
| 10 | Belgium | 3 | 151 | 3 | 4 | 0 |

Table S3. Top 10 co-cited references related to tumor microenvironment in non-small cell lung cancer with epidermal growth factor receptor mutation.

| NO. | Title | First Author | Journal | Year | Citations | IF(2023) | Centrality |
| --- | --- | --- | --- | --- | --- | --- | --- |
| 1 | Nivolumab versus Docetaxel in Advanced Nonsquamous Non-Small-Cell Lung Cancer | Borghaei H | New Engl J Med | 2015 | 52 | 96.2 | 0.08 |
| 2 | EGFR Mutations and ALK Rearrangements Are Associated with Low Response Rates to PD-1 Pathway Blockade in Non-Small Cell Lung Cancer: A Retrospective Analysis | Gainor JF | Clin Cancer Res | 2016 | 40 | 10 | 0.24 |
| 3 | Pembrolizumab versus docetaxel for previously treated, PD-L1-positive, advanced non-small-cell lung cancer (KEYNOTE-010): a randomised controlled trial | Herbst RS | Lancet | 2016 | 32 | 98.4 | 0.01 |
| 4 | Atezolizumab versus docetaxel in patients with previously treated non-small-cell lung cancer (OAK): a phase 3, open-label, multicentre randomised controlled trial | Rittmeyer A | Lancet | 2017 | 33 | 98.4 | 0.26 |
| 5 | Pembrolizumab versus Chemotherapy for PD-L1-Positive Non-Small-Cell Lung Cancer | Reck M | New Engl J Med | 2016 | 33 | 96.2 | 0.16 |
| 6 | Activation of the PD-1 pathway contributes to immune escape in EGFR-driven lung tumors | Akbay EA | Cancer Discov | 2013 | 27 | 29.7 | 0.06 |
| 7 | Nivolumab versus Docetaxel in Advanced Squamous-Cell Non-Small-Cell Lung Cancer | Brahmer J | New Engl J Med | 2015 | 26 | 96.2 | 0.02 |
| 8 | Cancer immunology. Mutational landscape determines sensitivity to PD-1 blockade in non-small cell lung cancer | Rizvi NA | Science | 2015 | 26 | 44.7 | 0.06 |
| 9 | Checkpoint Inhibitors in Metastatic EGFR-Mutated Non-Small Cell Lung Cancer-A Meta-Analysis | Lee CK | J Thorac Oncol | 2017 | 29 | 21 | 0.06 |
| 10 | Upregulation of PD-L1 by EGFR Activation Mediates the Immune Escape in EGFR-Driven NSCLC: Implication for Optional Immune Targeted Therapy for NSCLC Patients with EGFR Mutation | Chen N | J Thorac Oncol | 2015 | 18 | 21 | 0.06 |
